# Dissolution of Bovine Palatal Tissue with Degassed Sodium Hypochlorite

**DOI:** 10.3390/dj13030110

**Published:** 2025-02-28

**Authors:** Justin Fang, Zhen Shen, Ransome van der Hoeven, David E. Jaramillo

**Affiliations:** Department of Endodontics, School of Dentistry, University of Texas Health Science Center at Houston, Houston, TX 77054, USA; shzhenen@gmail.com (Z.S.); ransome.vanderhoeven@uth.tmc.edu (R.v.d.H.)

**Keywords:** GentleWave^®^, irrigation, tissue dissolution, NaOCl, endodontics

## Abstract

**Objective:** To investigate the tissue dissolution efficacy of different concentrations of sodium hypochlorite (NaOCl) using the GentleWave^®^ technology (GW) and compare it with that of passive ultrasonic irrigation. This study will provide a novel in vitro model used to evaluate the tissue dissolution efficacy of different concentrations of NaOCl using the GentleWave^®^ procedure. **Materials and Methods:** Palatal bovine tissue was standardized by weight at 70–75 mg (average of 74.4 mg). The samples were divided into three groups of *n* = 10, Group 1: GW + 0.5% NaOCl, Group 2: GW + 3% NaOCl, and Group 3: ultrasonic group (US) + 6% NaOCl, and a control group of *n* = 2 with GW + H_2_O. A specialized CleanFlow instrument was manufactured for the GW groups. For the US group, an apparatus was developed to allow the tissue to be in close contact with a freely operating ultrasonic tip. Each group was operated with its specific irrigants and dissolution times were obtained unless the samples were not visually dissolved within 5 min, and the dissolution rates were calculated. Dissolution times and rates were analyzed using univariate analysis of variance followed by a *t*-test. **Results:** The GW groups with 0.5% and 3% NaOCl dissolved entire tissue samples within 5 min and had average dissolution times of 227.5 s (Group 1) and 81.5 s (Group 2). GW with water had a higher tissue dissolution rate than ultrasonics with 6% NaOCl. **Conclusions:** The GentleWave^®^ using a lower concentration of NaOCl showed a similar clinical efficacy of dissolving tissues but displayed a significantly faster rate when compared with passive ultrasonic agitation.

## 1. Introduction

In endodontics, irrigation is often considered the most important aspect of treatment. Root canal treatments provide a mixture of mechanical, chemical and microbiological functions and is best accomplished with irrigation [1]. Without proper irrigation, the areas within the root canal system unaffected by mechanical instrumentation would remain untouched [1]. Irrigation has a multitude of purposes, including tissue dissolution of both organic and inorganic material and antimicrobial properties, while reducing friction and heat with files [1]. Endodontic irrigants can be best considered based on their antimicrobial capability, capacity of necrotic tissue dissolution, debridement ability, and toxicity to periradicular tissues [2].

Sodium hypochlorite (NaOCl) has become the most popular endodontic irrigant of choice due to its antimicrobial action, ability to dissolve tissue debris, and low cost [3,4]. Currently, it is the only clinically acceptable solution that can dissolve organic tissues [5]. On the other hand, ethylenediaminetetraacetic acid (EDTA) is commonly used to dissolve inorganic tissues [5,6]. A major concern about NaOCl, however, is the effects on periapical tissues due to its toxicity, which can contribute to postoperative pain [1]. Studies have found that lowering the concentration of NaOCl can lead to a significantly lower incidence and intensity of pain [7].

The effectiveness of NaOCl on organic tissue dissolution depends on the concentration used, and any concentration between 0.5% and 6% can be used successfully in clinical endodontics depending on the protocol [8,9]. Higher concentrations can lead to more harmful and continuing inflammatory reactions in the connective tissue as well as a higher potential for extrusion periapically [7,10]. As a result, patients may experience more postoperative pain [7]. On the other hand, lower concentrations of NaOCl, such as 0.5%, when used in greater volumes and with longer irrigation times, still possessed substantial bactericidal activity [11]. With concentrations even as low as 0.006%, NaOCl was able to eradicate Enterococcus faecalis in 1 min [12]. Utilizing conventional irrigation methods, lower concentrations of NaOCl still maintained considerable tissue dissolution capacity and cleaning capabilities of root canals [13].

All concentrations of NaOCl were shown to be equally effective in flushing out loose debris, pulpal remnants, and predentin from non-instrumented canal walls [14]. Necrotic pulpal tissue remnants were dissolved at a clinically similar rate between lower and full strength NaOCl concentrations. Practitioners can compensate for a lower NaOCl concentration by frequently replenishing the irrigant solution, and by using an enhanced debridement technique [8].

Utilizing irrigation adjuncts is an efficient and proven way to enhance the effectiveness of NaOCl. Agitation of NaOCl has been proven to enhance its tissue-dissolving activity [5,8,15]. Ultrasonic activation has been reported to aid in the dissolution of organic and inorganic debris during endodontic treatment through streaming and cavitation of the solution [16,17]. The main limitation has been that irrigants can only progress roughly 1 mm beyond the tip of the ultrasonic device. Hence, canals must be prepared to be large enough to accommodate the device close to the apical constriction to achieve maximum benefit [17,18].

The GentleWave^®^ (Sonendo Inc, Laguna Hills, CA, USA) is a novel irrigation system that was developed to further clean and disinfect the root canal system while conserving tooth structure when compared to traditional endodontic preparations [19]. The GentleWave^®^ handpiece requires a platform to be built for the best fluid exchange results and to minimize leakage. The GentleWave^®^ technology creates a strong hydrodynamic cavitation that uses degassed fluid and produces a large spectrum of sound waves within the fluid [19]. The degassed fluid consists of NaOCl, EDTA, and distilled water that has an overall reduced amount of dissolved gas so that its delivery is enhanced [20]. It was discovered that when using the GentleWave^®^ system, tissue dissolution efficacy was eight times greater than that of traditional endodontic irrigation systems [5,21].

The concentration of NaOCl used in root canal therapy can affect the biological response of the tissue and surrounding structures. High concentrations can lead to more destructive reactions in the connective tissue, which can lead to more frequent and severe postoperative complications for patients [7]. In this study, we provided a novel in vitro model used to evaluate the tissue dissolution efficacy of different concentrations of NaOCl using the GentleWave^®^ procedure. We hypothesized that the GentleWave^®^ device running with a lower concentration of NaOCl (0.5%) will result in a similar tissue dissolution efficacy clinically compared to that of the standard 3% solution and using passive ultrasonic irrigation.

## 2. Materials and Methods

NaOCl concentrations of 0.5%, 3% and 6% were tested. A stock solution of 6% NaOCl (Clorox Bleach; Clorox, Oakland, CA, USA) was obtained from the manufacturer. The solutions were brought to room temperature before use and diluted in bulk by pouring equal volumes of NaOCl and distilled water until the desired concentrations were obtained. As a negative control, distilled water from the GentleWave^®^ (GW) console was also tested as the irrigation of choice. The GentleWave^®^ console was set up according to the manufacturer’s instructions.

Palatal soft tissue samples from fresh bovine heads were cut into pieces of 2 × 2 × 2 mm using a sterile stainless-steel blade and standardized by ensuring the weight was between 70 and 75 mg (average of 74.4 mg) using a calibrated electronic balance. Before use, the samples were kept in phosphate-buffered saline (PBS) and frozen to −80 °C in standard packaged freezer test tubes to retain moisture. The samples were randomly divided into three groups of *n* = 10 each, Group 1: GW + 0.5% NaOCl, Group 2: GW 3% + NaOCl, and Group 3: Ultrasonic (US) + 6% NaOCl, and a control group of *n* = 2 with GW + H_2_O.

Samples were defrosted by removing the tubes from the freezer and leaving them at room temperature for 30 min before use, and excess moisture was dried with sterile gauze. The GW system consists of a console and a CleanFlow handpiece. The CleanFlow head fits in a platform that is built around the tooth with the manufacturer’s light-cured sealing solution to provide a tight seal. The system was operated from a touch screen control panel on the console that allows the user to regulate the concentration of irrigants. The control panel also sets the criteria for the procedure, including the tooth type, duration of irrigation, and type of solution to use. The irrigants flow from the central unit to the CleanFlow handpiece and then through the evacuation tubing that is connected to the handpiece back to the waste bucket in the central unit.

**Figure 1:**
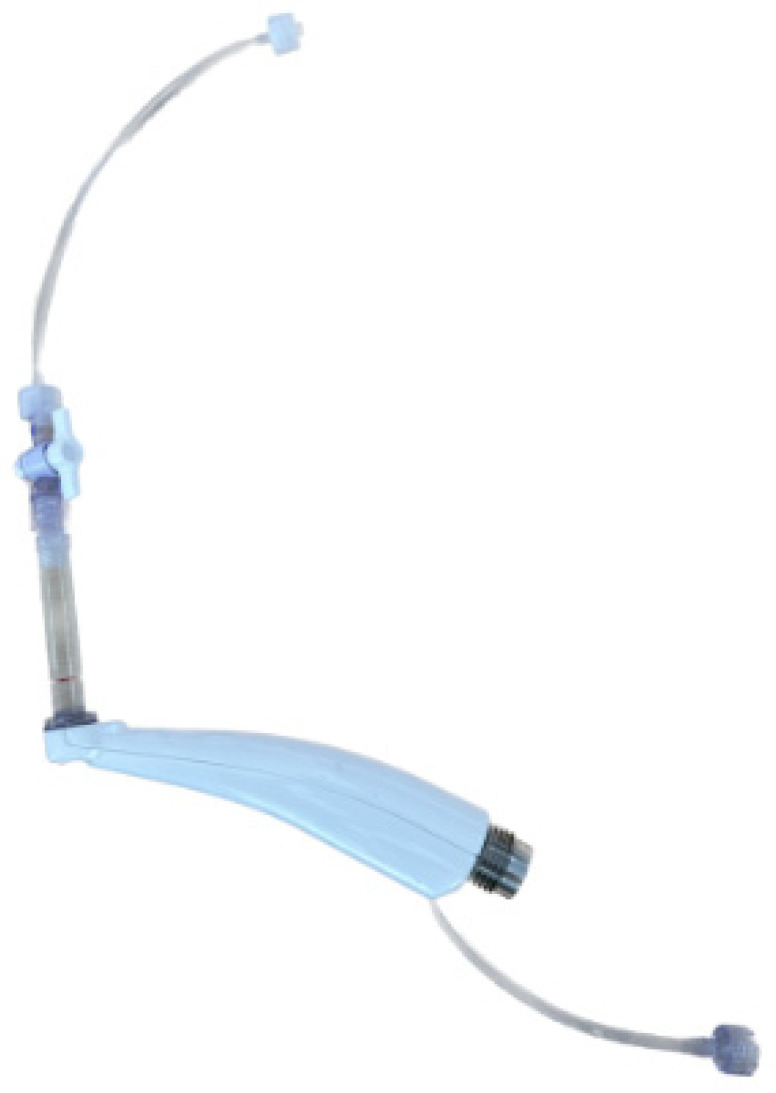
Specialized CleanFlow handpiece. Specialized Cleanflow handpiece with glass vial, mesh net, stopcock, and evacuation tubing.

In this particular in vitro model, a specialized CleanFlow instrument was manufactured with a glass vial and an attached mesh net (Figure 1). At the other end of the instrument, a stopcock was attached to the evacuation tubing that returns to the GW console to control the solution flow. The tissue was placed in a glass vial, while the mesh net prevented it from being suctioned through the evacuation tubing and into the waste. The GW system was run according to the manufacturer’s protocol, utilizing 0.5% and 3% NaOCl for the respective groups to obtain dissolution times. If the specimen was not visually dissolved within 5 min, the sample was dried and its weight was measured. Rate of dissolution was then calculated. If the specimen was completely dissolved within 5 min, the rate of dissolution was 100%.

For the ultrasonic group, a separate apparatus was used, consisting of a glass vial, consistent in size with that of the GW groups, that was stabilized to be flat on the tabletop. The bovine tissue was suspended in the middle of the vial by inserting a metal wire through the sample and stabilizing the wire to the outside of the vial to prevent movement. An Irrisafe^®^ (Satelec Acteon, Mérignac, France) tip was placed in the vial without contacting the specimen and used according to the manufacturer’s protocol, with the tip freely operating at 25% power. Full-strength NaOCl was placed in the vial and constantly replenished by hand with a syringe and 30-gauge needle in 1 min intervals. Dissolution times were obtained by visually determining whether the samples were dissolved within 5 min. If the specimen was not visually dissolved within 5 min, the sample was dried and the weight was measured. Rate of dissolution was then calculated. If the specimen was completely dissolved within 5 min, the dissolution rate was 100%.

The values for each device and concentration of NaOCl irrigant tested were expressed as tissue dissolution times. If the samples were visibly dissolved within 5 min, the time it took to dissolve the entire sample visibly was directly determined to be the dissolution time. If the specimens were not visually dissolved within the 5 min time frame, then the rates of tissue dissolution were calculated with the following formula:

Rate of Tissue Dissolution = (Percentage of Tissue Mass Loss)/(Recorded Time)

Percentage dissolved values were calculated by dividing the weight of the remaining sample by the average sample weight of 74.4 mg. The rates of tissue dissolution values were expressed as a mean rate of tissue dissolution ± standard deviation. Both tissue dissolution times and percent dissolved values were checked for normality with the Shapiro–Wilk test. The tissue dissolution times were compared using univariate analysis of variance followed by a *t*-test. The percentage dissolved values were determined to not be normally distributed, so non-parametric tests (Kruskal–Wallis and Dunn’s Multiple Comparison) were performed. Differences in mean tissue dissolution times and mean tissue dissolution rates were considered statistically significant if the *p* value was less than 0.05.

## 3. Results

The rate of tissue dissolution times and tissue dissolution rates using 0.5%, 3%, and 6% concentrations of NaOCl and distilled water with either the GentleWave^®^ or ultrasonic irrigation systems are shown in Table 1 and Table 2. The GW groups with both 0.5% and 3% NaOCl showed that the samples dissolved within 5 min and had average tissue dissolution times of 227.5 ± 57 s (Group 1) and 81.5 ± 18.9 s (Group 2). GentleWave^®^ with both 0.5% and 3% NaOCl as the irrigants dissolved the samples within the time interval of 5 min. The GW with 3% NaOCl group dissolved the sample almost 2.5 min faster than the GW with 0.5% NaOCl group (*p* < 0.01). Ultrasonic group 3 and the GW with water control group did not dissolve samples within the 5 min time frame, so tissue dissolution rates were expressed. The tissue dissolution rate was higher for the control group (33.5% ± 2.1%) than that of Group 3 (8.9% ± 18.1%) (*p* < 0.01). In other words, more of the samples dissolved with the GentleWave^®^ and distilled water as the irrigant compared with the ultrasonic and 6% NaOCl as the irrigant. Because the entire tissue sample was visually dissolved within 5 min for Groups 1 and 2, the tissue dissolution rate for those respective groups was considered 100%.

## 4. Discussion

Several different types of experimental designs have been used in previous research to measure tissue dissolution rates, with varying solutions of irrigation solutions and/or irrigation systems. This in vitro model showed that the entire tissue sample was dissolved within 5 min for both experimental GW groups (Groups 1 and 2) but not for the US and control groups (Groups 3 and 4). These results rejected our null hypothesis. Both GW NaOCl groups dissolved tissue within the 5 min NaOCl cycle. GW conducted with 0.5% NaOCl showed a similar tissue dissolution efficacy clinically compared to 3% NaOCl. However, GW conducted with distilled water dissolved more tissue within the 5 min time period compared with passive ultrasonic irrigation using 6% NaOCl.

It has been shown that a higher flow rate for irrigation alone cannot explain a more efficient tissue dissolution rate [5]. The GentleWave^®^ technology creates an intense hydrodynamic cavitation that uses degassed fluid and produces a large spectrum of sound waves within the fluid [19]. It has been reported that air in the irrigation fluid can cushion the implosive effects of cavitation. An implosion of microbubbles creates an acoustic field of broadband frequencies that is distributed throughout the fluids to reach the entire root canal system, which enhances soft tissue cleaning and bacteria elimination [20]. In effect, few to no bubbles are present in the irrigation fluid, which provides more constant contact with the tissue. This may lead to a more efficient tissue dissolution effect.

Clinically, using NaOCl as an irrigating solution can present a concern of extruding the solution outside the apical foramen. It has been shown that the GentleWave^®^ technology extrudes less irrigant apically compared to standard irrigation techniques due to its negative pressure system [22].

The limitations of the present study include the type of tissue used, the subjective nature of determining complete dissolution visually, and the vial size or geometry. Ideally, human necrotic pulp tissue should be used for this type of testing. However, due to limited availability and difficulty in obtaining it, bovine tissue was chosen because it has a fairly uniform composition and is easily available. When determining tissue dissolution time, the samples were deemed dissolved when they were visually gone, but there is a subjective component in this judgement. The sample may appear to be visually dissolved but it is possible there are tissue remnants that are not easily visible to the naked eye. High-resolution imaging or spectrophotometric analysis may be employed in the future to confirm the complete dissolution of tissues and eliminate observation bias. Lastly, the vial geometry and size could have been altered to reflect the different root canal sizes and shapes. This may affect the distance and path that irrigants must travel to dissolve the tissue.

This study has shown that lowering the concentration of NaOCl in the GentleWave^®^ system still produces a clinically similar tissue dissolution efficacy to the original concentration. Future studies should expand the sample size based on this pilot study and also evaluate the difference in frequency and severity of postoperative complications between 0.5% NaOCl and 3% NaOCl with GentleWave^®^. This will help determine the direct benefit of lowering the concentration of NaOCl with the GentleWave^®^ system.

Recent studies have shown that the GentleWave^®^ system could be an effective disinfection protocol in removing the biofilm, even in non-instrumented or minimally instrumented root canal systems [23,24]. In addition, the GentleWave^®^ system is very effective in removing lipoteichoic acid [25] and lipopolysaccharides [26] compared to passive ultrasonic irrigation systems. Therefore, with the modification of vial geometry and size to reflect the different root canal sizes and shapes, our newly designed in vitro system has the potential to be developed into a platform to study the effectiveness of biofilm and toxin removal. Furthermore, this platform may also be utilized to compare the efficacy of the GentleWave^®^ system with other adjunct disinfection protocols, such as the Er,Cr:YSGG laser, which has been proven effective [27,28,29].

## 5. Conclusions

The GentleWave^®^ technology using 0.5% NaOCl dissolved tissue at a clinically similar efficacy when compared with using 3% NaOCl, but still showed a significantly faster rate when compared with a passive ultrasonic irrigation system in this present study.

## Figures and Tables

**Table 1:** Percentage of tissue dissolution (*p* < 0.01).

	GentleWave^®^ 0.5% NaOCl	GentleWave^®^ 3% NaOCl	Ultrasonic 6% NaOCl	GentleWave^®^ Distilled Water
Sample Size	10	10	10	2
Mean (% dissolved)	100	100	8.87	33.51
STD	0	0	18.07	2.12

**Table 2:** Dissolution time for the 2 GentleWave^®^ groups (*p* < 0.01).

	GentleWave^®^ 0.5% NaOCl	GentleWave^®^ 3% NaOCl
Sample Size	10	10
Mean (Time in seconds)	227.5	81.5
STD	57.95	18.92

## Data Availability

The original contributions presented in the study are included in the article, further inquiries can be directed to the corresponding authors.
